# Using Simulation Optimization to Solve Patient Appointment Scheduling and Examination Room Assignment Problems for Patients Undergoing Ultrasound Examination

**DOI:** 10.3390/healthcare10010164

**Published:** 2022-01-15

**Authors:** Ping-Shun Chen, Gary Yu-Hsin Chen, Li-Wen Liu, Ching-Ping Zheng, Wen-Tso Huang

**Affiliations:** 1 Department of Industrial and Systems Engineering, Chung Yuan Christian University, Chung Li District, Taoyuan City 320314, Taiwan; pingshun@cycu.edu.tw (P.-S.C.); liuliwen.iem08g@nctu.edu.tw (L.-W.L.); minnie8668@gmail.com (C.-P.Z.); 2 Department of Logistics Management, National Kaohsiung University of Science & Technology, Yanchao District, Kaohsiung City 82445, Taiwan; garychen@nkust.edu.tw; 3 Department of Business Administration, Chung Yuan Christian University, Chung Li District, Taoyuan City 320314, Taiwan

**Keywords:** patient appointment scheduling, radiological technologist, simulation optimization, system simulation, ultrasound examination, workload balance

## Abstract

This study investigates patient appointment scheduling and examination room assignment problems involving patients who undergo ultrasound examination with considerations of multiple examination rooms, multiple types of patients, multiple body parts to be examined, and special restrictions. Following are the recommended time intervals based on the findings of three scenarios in this study: In Scenario 1, the time interval recommended for patients’ arrival at the radiology department on the day of the examination is 18 min. In Scenario 2, it is best to assign patients to examination rooms based on weighted cumulative examination points. In Scenario 3, we recommend that three outpatients come to the radiology department every 18 min to undergo ultrasound examinations; the number of inpatients and emergency patients arriving for ultrasound examination is consistent with the original time interval distribution. Simulation optimization may provide solutions to the problems of appointment scheduling and examination room assignment problems to balance the workload of radiological technologists, maintain high equipment utilization rates, and reduce waiting times for patients undergoing ultrasound examination.

## 1. Introduction

Hospitals are a service industry; therefore, enhancing quality and efficiency is a key factor in enhancing competitiveness. Most customers value efficiency and judge hospitals on the quality of their services and the fluency of service flow—the occurrence of waiting is a leading target for complaints [1,2,3]. Efficiency can be enhanced if patients feel they have saved a notable amount of time. In a large-scale study of patients during October 2014–February 2017, Sun et al. [4] found that patients’ waiting times negatively correlated with their satisfaction with the hospital. Therefore, improving patient appointment scheduling and the manner in which patients are assigned to examination rooms (or clinical rooms) are crucial [1,5,6,7,8,9]. The literature discusses patient appointment scheduling in hospitals and methods for assigning patients to examination rooms [1,5] but not how these affect patient waiting times and the workload of radiological technologists. Furthermore, certain scholars applied methodologies from various academic disciplines, such as system dynamics [10,11], finite machines [12], or Six Sigma [13] to investigate the dropout or overcrowded conditions in hospitals. Therefore, this study not only investigates appointment scheduling for patients undergoing ultrasound examination, but also explores the methods for assigning these patients to examination rooms. In addition, most studies focus on improving equipment utilization rates and reducing patient idle times [1,5,14], whereas this study includes a discussion on the fairness of workload distribution among radiological technologists.

In studies regarding patient appointment scheduling, the type of patients diagnosed typically consists of a single type [15,16], such as otorhinolaryngology or ophthalmology, and the patient visit time is assumed to be a single probability distribution [6,17,18,19,20]. Additionally, patients in this study were subjected to external examinations by radiologists; if internal examinations were required, specialists other than radiologists would take care of the patients. However, patients in a radiology department may be referred from various other departments, such as the thoracic department and the hematology oncology department. Moreover, the body parts to be examined differ according to patient conditions, such as scanning the limbs, abdomen, or chest. Scanning different body parts may result in different time probability distributions [1,8]. The literature also only discusses single clinical rooms, whereas the radiology department of the hospital investigated in this study has multiple examination rooms. The use of multiple examination rooms—including nuclear magnetic resonance, tomography and ultrasound examination—is more commonly seen in hospitals. Ultrasound examinations in the radiology department require a radiological technologist to operate ultrasound probes and come into contact with a patient’s body. When a patient’s intimate body parts, such as the prostate or scrotum, or those with deep vein thrombosis, require examination, the procedure must be carried out by a radiological technologist of the same sex as the patient to prevent any inappropriate scenarios. Therefore, this involves assigning radiological technologists to examination rooms. Existing studies do not discuss this limitation [1,6,8], but this study includes this sex-based limitation on radiological technologists when solving the problem of assigning patients to examination rooms.

The objective of this study is to construct a simulation model to simulate the ultrasound examination process in the case hospital, and use simulation optimization to investigate the better appointment time interval under the conditions of multiple types of patients, multiple examination rooms, multiple scanned body parts of patients, and multiple radiological technologists. Hence, this study provides better solutions for assigning patients undergoing ultrasound examination to examination rooms, thereby balancing the workload of radiological technologists, maintaining high equipment utilization rates, shortening waiting time for patients, and eventually reaching a mutually beneficial situation for radiological technologists, the hospital, and patients.

The remaining parts of this paper are organized as follows. The existing research on this research topic is discussed in Section 2. Section 3 presents the proposed research procedure. Section 4 provides the simulation of a case study and also the verification and validation of the results from the simulation model. Finally, Section 5 concludes this study and points out future directions of this research.

## 2. Literature Review

The literature surveyed in this study mainly relates to appointment scheduling for patients undergoing ultrasound examination. The patient appointment scheduling problem involves variables such as number of physicians and examination rooms (single or multiple rooms), type of patients (single or multiple types), patient visit-time distribution (constant or random arrival time), and patient examination-time intervals (fixed and variable time interval) and considers whether a patient arrives on time or not [14,21], and whether a patient is absent [14,22,23,24]. Methods such as system simulation [15,25], simulation optimization [1,17,26,27,28], heuristic or meta-heuristic algorithms [6,16,23,29], and mathematical programming [5,19,30] are employed to solve the patient appointment scheduling problems. This study reviews the literature associated with these problems. Specifically, the research question of this study involves investigating patient appointment scheduling problems concerning multiple types of patients (outpatients, inpatients, and emergency patients), multiple body parts to be examined, and special limitations on the ultrasound examination operation (e.g., intimate body parts must be examined by a radiological technologist of the same sex as the patient) under the condition that multiple examination rooms are available. The patient appointment scheduling problem investigated in this study differs from that studied by other scholars, and this difference is the study’s main motivation.

### 2.1. Patient Appointment Scheduling

Patient appointment scheduling can be seen as a resource scheduling problem with uncertainty; resource scheduling has developed several variations, such as surgical scheduling problems in the open room surgery area [29,31,32,33,34,35,36,37]. Baril et al. [38] studied the appointment schedules for orthopedic outpatients by investigating three types of outpatients and different visiting times. They decided how to allocate patient appointment schedules and placement of nursing staff in three examination rooms to maximize physician utilization, minimize patient waiting times, and maximize patient examinations. Bhattacharjee and Ray [39] introduced the patient flow pattern involving the allocation uncertainty of patients’ check-in time, their absence, the diversion rate of patients’ visiting procedures, and patients’ visiting time or examination time. With regard to performance measures, they surveyed different types of visiting procedures including emergency rooms, operating rooms, inpatient care, and diagnostic facilities. For this research related to patient appointment scheduling and exam room assignment at a radiological department, discrete event simulation is most suitable because this problem matches the characteristics of discrete event simulation best: (1) complex patient flows (2) transient analysis of system performance, and (3) a scheduling and resource allocation-related problem. Wu et al. [40] studied the minimization of total patient waiting times and total idle time in a scenario where patients waited in line following a first-in-first-out approach to be examined at one of three examination rooms. Chen et al. [1] compared the traditional fixed patient number appointment scheduling strategy and the mixed patient number appointment scheduling strategy. They developed a patient number appointment scheduling strategy and an irregular patient number appointment scheduling strategy.

Huang and Marcak [41] considered new patients and returning patients to organize a decision tree to determine the minimum grid point value based on different results. Huang [42] considered the patients’ no-show rate to investigate the corresponding costs. The simulation results indicated the lowest examination room cost existed if the patient population was assigned to four categories. Liu [43] proposed the waiting window at the time of appointment through mathematical inference which suggests in the limited waiting space, decide on the appropriate patient appointment window in order to maximize compensation. Ma et al. [44] investigated—through system simulation and simulation optimization—the effects of different new patient appointment times and patient visit intervals on physician workload and examination room capacity. Gavriloff et al. [45] found that using load balancing allowed more outpatients to access care more quickly under the same medical funding. Huang et al. [20] introduced the drum-buffer-rope (DBR) scheduling approach to analyze which type of medical resource was the bottleneck resource for optimizing operating room scheduling. After verifying the effectiveness of the DBR method in uncertain situations, a Monte Carlo simulation was executed. Deceuninck et al. [21] proposed a control variate method for scheduling with unpunctual patients. Their method considerably improved the precision of the simulation estimates through the judicious use of control variates compared with the Monte-Carlo simulation.

### 2.2. Hospital System Simulation

Zeltyn et al. [46] carried out a system simulation to construct a pull-out emergency room system and calculated the resource workload and the average workload per resource to observe the nurse utilization of each group. Masselink et al. [47] researched the waiting line system combined with the system simulation method to explore the planning strategy of chemotherapy drugs to minimize the waste cost. The results showed that the approach of medicine prepared in advance did reduce patient waiting times, but it also slightly increased the cost of drug waste. Rau et al. [18] adopted the system simulation as the research methodology to explore different types of rehabilitation resource allocation situation. Ferrand et al. [48] used Arena system simulations at different patient arrival rates and different surgical times to analyze different situations and various performance results to find a better operating room configuration. Devapriya et al. [49] used a system simulation to predict patient numbers and the length of stay in hospitals to determine their corresponding performance on bed utilization rate, patient waiting time, and cost. The results could be provided to hospital managers for future hospital bed expansion planning reference. Mallor et al. [50] analyzed the performance of different patient treatment times based on different dispatching rules, and then compared the advantages and disadvantages of mathematic and simulation results according to the examination room data.

Rico et al. [51] utilized Arena system simulations to construct the medical process of case hospitals. They hypothesized that the arrival rate of influenza patients to the hospital within 12 weeks was a Poisson distribution, while the medical process time followed a triangular distribution. The nursing and medical resources were optimally configured at various degrees of isolation similar to patient login classification areas, e.g., green, yellow, red, and black areas. Ramis et al. [52] adopted the Flexsim system simulations and found that nurses had the least waiting time in the system if they were given a certain level of attention during peak hours to patients with and without appointments. Ahmed and Alkhamis [17] have shown that the developed analog optimization heuristic algorithm was better than the current hospital option, for patients in three categories, with the average waiting time reduced by 22%, 45%, and 53%, respectively, while the number of patients completing surgery per hour increased by 28%. Klassen and Yoogalingam [26] analyzed the sensitivity of the developed analog optimization heuristic algorithm according to the different parameters of five scheduled patients, three kinds of surgeons’ idle cost, four kinds of patient no-show rate, and two kinds of outpatient length of period per physician.

Asgary et al. [53] used system simulation to construct a drive-through mass vaccination model to determine the appropriate medical resources for reducing drive-through patients’ waiting time. Wu et al. [54] used the AnyLogic agent-based simulation tool to construct an emergency evacuation plan for the case hospital in order to better allocate medical staff to reduce the patient’s evacuation time. Zou et al. [55] introduced the priority and avoidance rules into the modified cellular automata model. The results showed that the routes of wheelchairs were shortened and both waiting and evacuation time were significantly reduced due to the priority setting. Tamburis and Esposito [56] showed how the knowledge of process mining techniques could provide a robust premise to build a discrete event simulation model of a healthcare process.

### 2.3. A Summary

For the patient appointment scheduling problem, many researchers have applied mathematical programming to formulate their patient appointment scheduling models [5,6,8,14], and have developed different heuristic algorithms [5,8,14] or meta-heuristic algorithms [6] to achieve near-optimal solutions. To account for the uncertainty of patient arrivals or physician treatment/patient examination times, some scholars have used stochastic programming to build their mathematical models [5,14,19,35]. Furthermore, researchers have used system simulations in research on patient appointment scheduling problems [1,17,18] with real data from hospitals as input because they save time and are highly cost-effective. Management may later use the findings from these simulated scenarios to develop better policies. For the examination room assignment problems, researchers have used system simulation to compare the performance of different examination room assignment policies [1,6]. Based on the literature review, this study summarizes the related outpatient appointment scheduling and operating room scheduling papers in Table 1.

For this study, we investigate a patient appointment scheduling problem and an examination room assignment problem involving patients who undergo ultrasound examination with considerations of multiple examination rooms, multiple types of patients, multiple body parts to be examined, and special restrictions. For the proposed integrated problem it is very complicated to build up its corresponding mathematical model based on the rolling time periods. Therefore, this study uses system simulation to develop an investigated problem model, and uses simulation optimization to optimize the model. A detailed description of the research method and procedures are introduced in Section 3.

## 3. Methodology

### 3.1. Research Problem

The research problem of this study concerns comparison of the performance of different simulation models in three scenarios of reducing patients’ waiting time on their ultrasound examination day, balancing the workload of radiological technologists, and maintaining high equipment utilization rates. The scope of the research problem in this study involves a patient appointment scheduling problem under the conditions of multiple types of patients undergoing ultrasound examination, multiple examination rooms, and multiple body parts to be examined, and the problem of assigning patients to examination rooms. Different types of patients have different arrival time distributions. To simplify the complexity of the research problem, each ultrasound examination room is assumed to have testing equipment with the same functions. Furthermore, examination of different body parts results in different examination time distributions. By considering these scenarios, this study constructs a better appointment scheduling system for patients undergoing ultrasound examination, assigning them to examination rooms, finding a better time interval, and proposing a better method for assigning these patients. The research problem in this study has a number of limitations. A patient undergoing scrotal and prostate ultrasound examinations must be examined by a male radiological technologist, whereas a patient undergoing deep vein thrombosis ultrasound examination must be examined by a radiological technologist of the same sex. The hospital investigated in this study has six examination rooms (i.e., Examination Rooms 5 to 10). Moreover, according to the case radiology department’s rule, inpatients are given priority to undergo ultrasound examination in Examination Room 5, whereas emergency patients are given priority to undergo ultrasound examination in Examination Room 6.

This study determines a better time interval for the arrival of outpatients undergoing ultrasound examination. Time intervals for the arrival of inpatients and emergency patients undergoing ultrasound examination were mainly collected from the data provided by the hospital investigated in this study. This study explores a better method for assigning patients who have registered at the counter of the radiology department to examination rooms under the constraints of the hospital.

### 3.2. Research Procedure

The research procedures in this study were as follows: first, interviews were conducted with radiological technologists and appointment schedulers working in ultrasound examination rooms at the radiology department of the case hospital to understand the process of appointment scheduling for patients undergoing ultrasound examination and the data regarding time intervals for the arrival of various types of patients and the time taken to perform ultrasound examination on different body parts were collected. Based on the data collected, data fitting was used to determine corresponding probability distributions.

Second, this study involved the construction of a simulation model, verifying whether its performance differed from that of the actual system. If the results of statistical tests are significant, the simulation model must be corrected. If the results of statistical tests are insignificant, the simulation model can replace the actual system to perform subsequent investigations into the appointment scheduling time interval for patients undergoing ultrasound examination. This study uses the Equations (1)–(3) below to determine the number of replications (*n*) of the constructed simulation model. In Equation (1), $\bar{X}$ and *s* represent the average and standard deviation (SD) of the number of patients entering the system at the 95% (i.e., *α* = 95%) confidence interval, respectively. In Equation (2), *h*_0_ represents the initial half-width of the 95% confidence interval obtained in Equation (1). Equation (3) is used to calculate *n*, whereas *n*_0_ and *h* represent the initial number of replications and the adjusted half-width of the 95% confidence interval, respectively. A numerical example will be introduced in Section 4.4.

(1) $$The\;95\%\;confidence\;interval=[\bar{X}-t_{n-1,\;1-\frac{\alpha }{2}}\times \frac{s}{\sqrt{n}},\;\bar{X}+t_{n-1,\;1-\frac{\alpha }{2}}\times \frac{s}{\sqrt{n}}]$$

(2) $$h_{\;0}=t_{n-1,\;1-\frac{\alpha }{2}}\times \frac{s}{\sqrt{n}}$$

(3) $$n\approx n_{\;0}\times \frac{h_{\;0}^{\;2}}{h^{\;2}}$$

Finally, this study used simulation optimization, Arena with OptQuest, to identify a better appointment scheduling time interval for patients undergoing ultrasound examination according to the performance of the case hospital and explored improved methods for assigning patients to examination rooms in different scenarios, providing a reference for relevant decision-makers in the hospital. Arena’s OptQuest toolbox can identify the best possible outputs from input data to seek out, regulate, and evaluate. As a result, this study used the Arena simulation software to construct an optimized simulation model, and the OptQuest to determine the best appointment time interval that met the needs of the case hospital. OptQuest was also used to determine the best appointment scheduling time interval for multiple rooms, multiple types of patients, and ultrasound examination of multiple body parts. After the 10th simulation or iteration, OptQuest found the best solution among the feasible solutions presented by the algorithm. Computations were performed on an Intel Core i5-6200U 2.30 GHz PC (ASUS, Taipei city, Taiwan) with 8.00 GB of memory. Each simulation ran in less than 10 min.

### 3.3. Appointment Scheduling Process for Patients Undergoing Ultrasound Examination

**Figure 1:**
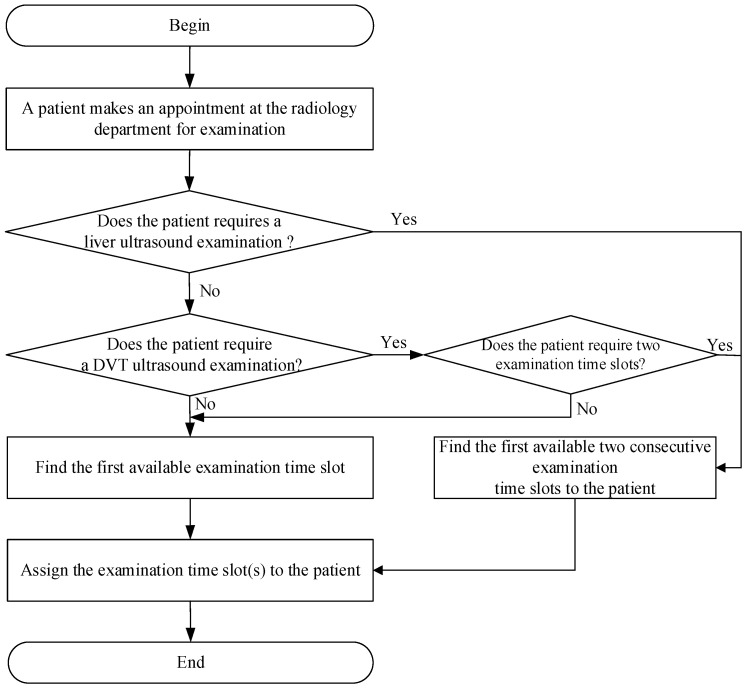
The flowchart of patient’s appointment procedure at the radiology department.

If a patient needs to schedule an ultrasound examination appointment after visiting a medical specialist, the patient must bring an examination appointment form to the counter at the radiology department to schedule the ultrasound examination, as shown in Figure 1. First, the appointment scheduler for the ultrasound examination determines whether the patient requires a liver ultrasound examination. If they do, the patient is then assigned to the first two consecutive available examination time slots. Otherwise, the scheduler determines whether the patient requires a deep vein thrombosis (DVT) ultrasound examination. If the person needs the DVT ultrasound examination, the scheduler determines whether the patient requires two examination time slots. If two examination time slots are required, the patient is then assigned to the first two consecutive available examination time slots. Otherwise, the patient will be assigned to the first available examination time slots. Moreover, if the patient requires an ultrasound examination of body parts other than the liver or deep vein thrombosis, the patient will be assigned to one of the first available examination time slots. The patient leaves the hospital after the person books an examination appointment.

The ultrasound examination at the case hospital has several limitations, including the limited number of male and female radiological technologists in each examination room, a cap on the number of examination points accrued by radiological technologists, and the size of the examination rooms. First, the male-to-female ratio of radiological technologists in each examination room, according to the actual shift schedule at the radiology department, is determined based on six-month data. Approximately 40% of the examination rooms have a male-to-female radiological technologist ratio of 6:6, which is the most common scenario. Therefore, the simulation system in this study assumes that the male-to-female radiological technologist ratio in each examination room is 1:1. In this study, male radiological technologists are assigned to Examination Rooms 6, 8, and 10, whereas female radiological technologists are assigned to Examination Rooms 5, 7, and 9. Regarding the size of the examination rooms, Examination Rooms 5 and 6 are more spacious than other examination rooms in the radiology department of the hospital. Moreover, inpatients and emergency patients are more likely to require hospital beds when undergoing ultrasound examination. Therefore, inpatients are given priority to be assigned to Examination Room 5, whereas emergency patients are given priority to be assigned to Examination Room 6.

### 3.4. Examination Room Assignment for Patients Undergoing Ultrasound Examination

**Figure 2:**
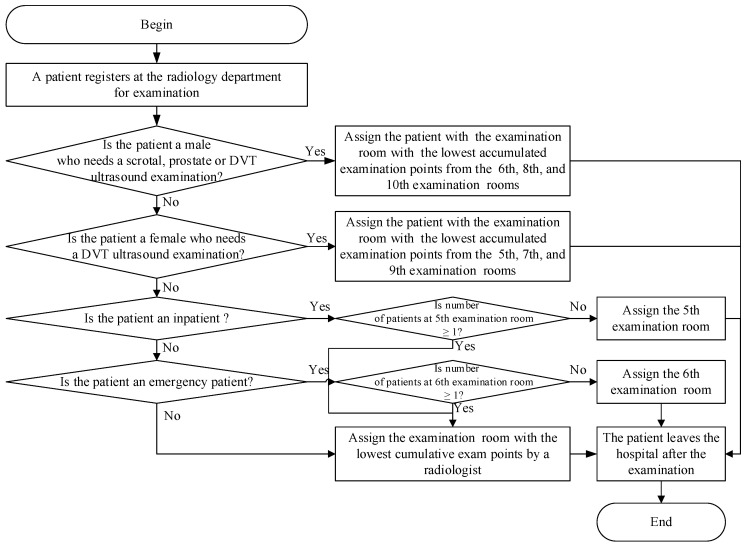
The flowchart of patient-examination room assignment at the radiology department.

Figure 2 showed the flowchart of the patients’ room assignment strategy devised by the appointment schedulers at the radiology department. Patients undergoing ultrasound examination at the hospital must go to the registration counter at the radiology department for registration on the examination appointment date. After the counter staff scans the appointment form, the system determines whether the patient is a male patient undergoing a scrotal or prostate ultrasound examination or whether the patient is a male patient undergoing a DVT ultrasound examination. If the patient requires either of those examinations, the patient is assigned to the examination room with the radiological technologist who has accumulated the lowest number of examination points out of Examination Rooms 6, 8, and 10. After the patient’s ultrasound examination is completed, the patient leaves the examination room.

If none of those examinations are required, the system determines whether the patient is a female patient undergoing a DVT ultrasound examination. If the answer is yes, the patient is assigned to the examination room with the radiological technologist who has accumulated the lowest number of examination points out of Examination Rooms 5, 7, and 9. After the ultrasound examination is completed, the patient leaves the examination room. Otherwise, the system determines whether the patient is an inpatient undergoing an ultrasound examination. If yes, the system continues to determine whether at least one patient is waiting in Examination Room 5. If the answer is yes, the patient is assigned to the examination room with the radiological technologist who has accumulated the lowest number of examination points out of all the examination rooms. Otherwise, the patient is assigned to Examination Room 5.

If the patient is not an inpatient, the system determines whether the patient is an emergency patient. If the person is an emergency patient, the system continues to determine whether at least one patient is waiting in Examination Room 6. If the answer is yes, the patient undergoing the ultrasound examination is then assigned to the examination room with the radiological technologist who has accumulated the lowest number of examination points out of all the examination rooms. Otherwise, the patient is assigned to Examination Room 6. If the patient is not an emergency patient, the patient is assigned to the examination room with the radiological technologist who has accumulated the lowest number of examination points out of all the examination rooms. The patient leaves the examination room after the ultrasound examination is complete.

## 4. Analysis and Discussion

### 4.1. Background of Case Study

The participants of this study were outpatients, inpatients, and emergency patients who underwent ultrasound examination in Examination Rooms 5 to 10 at the radiology department of the case hospital based on six-month data. Except for the breast ultrasound examination, the ultrasound examinations of all other body parts were conducted in Examination Rooms 5 to 10. One ultrasound machine was present in each examination room, and all the six ultrasound machines had the same functionalities. An examination appointment date was scheduled after a patient visited a physician and registered at the counter of the radiology department. The assignment to examination rooms for patients undergoing ultrasound examination was carried out on the examination day.

### 4.2. Data Regarding Patients Undergoing Ultrasound Examination

This study differentiated data regarding three types of patients undergoing ultrasound examination, namely outpatients, inpatients, and emergency patients. Because the examination policies at the hospital’s radiology department were different on Saturdays from those on weekdays, this study only investigated the examination policies applied on weekdays for six months, and deleted data regarding ultrasound examinations conducted on Saturdays, thereby ensuring that the data could be referenced. Table 2 illustrates that most of the patients undergoing ultrasound examination were outpatients, amounting to 10,133 people and accounting for 72% of the total number of patients, followed by inpatients and emergency patients.

Only patients undergoing scrotal, prostate, and DVT ultrasound examinations required radiological technologists of the same sex. Patients undergoing prostate and scrotal ultrasound examinations were only males, and the ratio of male to female outpatients undergoing DVT examination was 3:4. Under the fixed examination appointment scheduling strategy, a patient undergoing DVT ultrasound examination was allocated a fixed 20- or 40-min examination time according to the body parts to be examined when making an ultrasound examination appointment. However, a patient undergoing the liver ultrasound examination was allocated a 40-min examination time when scheduling an examination appointment date. A patient undergoing an ultrasound examination of body parts other than those two aforementioned body parts was allocated a fixed 20-min examination time.

### 4.3. Data Fitting Analysis

In this study, the data fitting analysis was performed using the Arena Input Analyzer developed by Rockwell Automation Inc (Rockwell Automation, Coraopolis, PA, USA). Chi-square and Kolmogorov-Smirnov (K-S) tests were conducted to find an appropriate probability distribution. If the data set passed the Chi-square and K-S test, an appropriate probability distribution was obtained. Otherwise, the number of data categories needed to change and data fitting analysis was performed again. If the data set passed the Chi-square and K-S tests after the number of data categories was changed, an appropriate probability distribution was obtained. If the set of data still failed to pass these tests, the hybrid distribution [56] and empirical distribution tests [57] were conducted on the data set, and the optimal distribution was selected as the appropriate probability distribution.

In this study, the data fitting analysis was conducted to find the appropriate probability distribution. Using data from outpatients undergoing prostate ultrasound examination as an example, the number of data categories was changed because this set of data failed to pass the Chi-square and K-S tests during the initial data fitting analysis, but no appropriate probability distribution was obtained. Therefore, hybrid and empirical distribution tests were performed on this data set, with hybrid distribution, 0.485 × (3 + 7 × BETA(1.9, 1.16)) + 0.424 × (10 + WEIB(3.46, 1.23)) + 0.091 × (20 + WEIB(6.99, 1.29)), selected as the appropriate probability distribution. Here, BETA and WEIB refer to Beta distribution and Weibull distribution, respectively.

### 4.4. Simulation Assumptions

**Figure 3:**
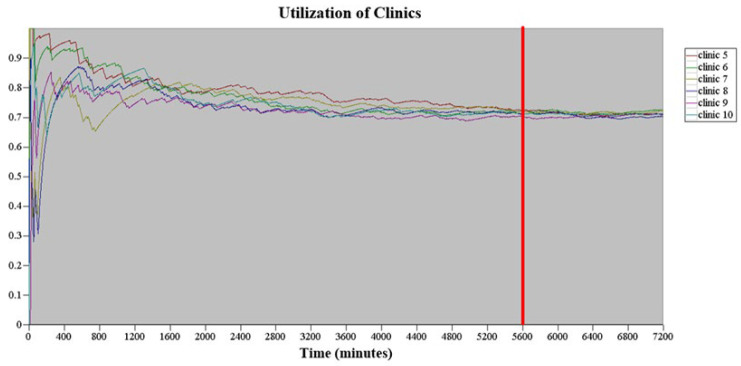
The warm-up period of the simulation model in this study.

This study involved nine simulation assumptions:

(1) Extremely small body parts were excluded from this study.
(2) There was no degradation in service quality or time provided to patients.
(3) We assumed that patients had received proper information during pre-examination procedures.
(4) This study only considered a particular time period of the ultrasound examination schedule, namely Monday to Friday from 8 am to 5 pm, with a break from 12 noon to 1 pm, totaling 8 h a day. The ultrasound examination time for outpatients, inpatients, and emergency patients regardless of the body parts accounted for 6.8% of the total examination time. Therefore, the actual daily simulation time was 93.2% of the total examination time, that is, 447.36 min (480 × 93.2%). The replication length of each simulation was 5 d (i.e., 2236.8 min). The simulation model in this study operated for 7200 min before analyzing the equipment utilization rate under steady-state conditions, as shown in Figure 3. When the simulation time reached 5600 min, no significant fluctuation was found in the equipment utilization rate. Therefore, the warm-up period of the simulation model in this study was set at 5600 min, and the replication length was 7836.8 min (5600 + 2236.8).
(5) The simulated examination rooms were Examination Rooms 5 to 10, totaling six examination rooms. No difference was assumed to exist among the examination rooms, and the instruments and equipment available in all the examination rooms were identical. Furthermore, all types of ultrasound examinations could be performed in each examination room. The same probability distribution was used for the examination time for patients undergoing ultrasound examination of the same body parts in different examination rooms.
(6) The examination times allocated by the ultrasound examination scheduler to patients undergoing liver and DVT ultrasound examinations were different from those allocated to patients undergoing ultrasound examination of other body parts. Based on the historical data regarding patients’ examinations, the examination time for 70% of patients undergoing DVT ultrasound examination was 20 min, whereas the examination time for 30% of patients undergoing DVT ultrasound examinations was 40 min. The examination time for patients undergoing liver ultrasound examination was 40 min, and the examination time for patients undergoing other ultrasound examination was 20 min.
(7) To develop an appointment scheduling strategy that balanced the workload of radiological technologists, the ultrasound examination room team leader assigned various points for different workloads, where 1 point represented 20 min and 2 points represented 40 min. For example, the workload for handling 30% of patients undergoing DVT ultrasound examination was equivalent to 2 points.
(8) The simulation model in this study assumed that the male-to-female radiological technologist ratio in the six examination rooms was 1:1.
(9) This study initially set the number of replications to 30, and used the number of patients undergoing ultrasound examination who entered the system as an indicator of the number of replications. When the initial number of replications, *n*_0_, was set to be 30, the average ($\bar{X}$) and standard deviation (SD) (*s*) of the number of patients entering the system at the 95% (i.e., *α* = 95%) confidence interval was obtained based on Equation (1) of the 30 replications, whereas the initial half-width (*h*_0_) was calculated based on Equation (2).

The average total number ($\bar{X}$) of outpatients entering the system was 351.7, whereas *h*_0_ was 6.1. The coefficient of variation (CV) value for outpatients was calculated by the half-width value divided by the average value, which was 1.73% and less than 0.02. However, the CV values for both inpatients and emergency patients were greater than 0.02. Therefore, to be more precise, this study set the CV value for outpatients to less than 0.01, and reduced the CV value for inpatients and emergency patients.

Equation (3) was used to calculate the number of replications required (*n*), whereas the adjusted half-width (*h*) was calculated based on the simulation results [58]. After the calculation of Equation (3), the CV value for outpatients was less than 0.01 when the adjusted number of replications should be greater than 89. Therefore, the number of replications in this study was increased to 110 to reduce the CV value for outpatients to less than 0.01. The average total number of outpatients entering the system was 353.3, and h was 3.5. With the CV value for outpatients being 0.0099, the CV value was reduced for both inpatients and emergency patients, for meeting the requirements of this study. For the subsequent simulation, the number of replications was set to 110 to ensure the stability of the simulation model.

### 4.5. Waiting Time Analysis for Patients Undergoing the Ultrasound Examination

No data was available regarding the actual time of entry of a patient into the examination room in the case hospital. The data was only available regarding the time between a patient’s registration of ultrasound examination and the upload of the first ultrasound image, as well as the time between the upload of the first and last ultrasound images. The actual waiting time for patients undergoing ultrasound examination was determined based on the duration from their registration to their entry into the examination room, and the actual time taken by the patients for using the examination room was the duration between their entry into the examination room and the upload of the last ultrasound image.

To construct the simulation model, it was necessary to determine the time between a patient’s entry into the examination room and the upload of the first ultrasound image to ensure consistency between the simulation model and the actual situation. Rico et al. [51] indicated that patient treatment time was characterized by randomness, and that minimum, mode, and maximum were data that could be collected easily. Thus, a triangular distribution was often applied to patient treatment (or examination) time.

In this study the radiological technologists and the ultrasound examination room team leader were asked to estimate the time between a patient’s entry into the examination room and the upload of the first ultrasound image and assumed that the time duration between a patient’s entry into the examination room and the upload of the first ultrasound image forms a triangular distribution. This study tested multiple time durations that exhibited the triangular distribution, and discovered that the distribution most consistent with the actual situation was as follows: minimum = 1 min, mode = 5 min, and maximum = 9 min. According to the simulation model, the time between a patient’s entry into the examination and the upload of the first ultrasound image was approximately 5 min, and the waiting time was approximately 7.2 min. The sum of both values was the same with the waiting time calculated based on the data available at the radiology department of the case hospital, which was 12.2 min. Thereafter, the triangular distribution—TRIA (1, 5, 9)—was used as the time between a patient’s entry into the examination room and the upload of the first ultrasound image in this study.

### 4.6. Verification and Validation of the Simulation Model

This study confirmed the correctness of the constructed simulation model (verification) and whether its performance was consistent with that of the actual system (validation)—that is, the validity of the simulation model—by using the data provided by the case hospital. The procedures of verification and validation were as follows:

(1) Verification

**Figure 4:**
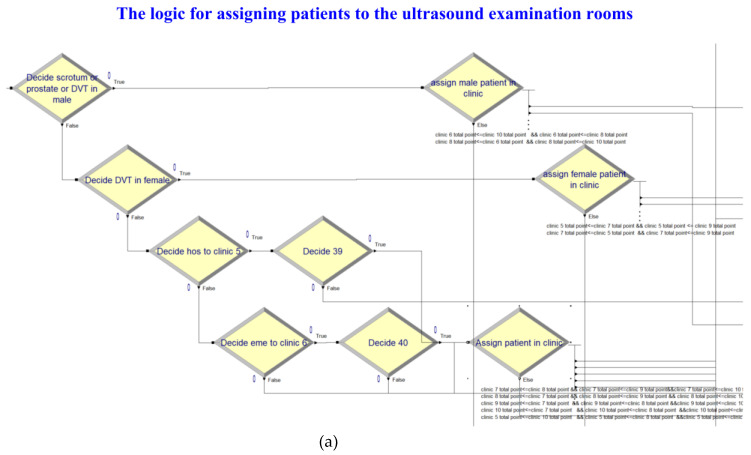

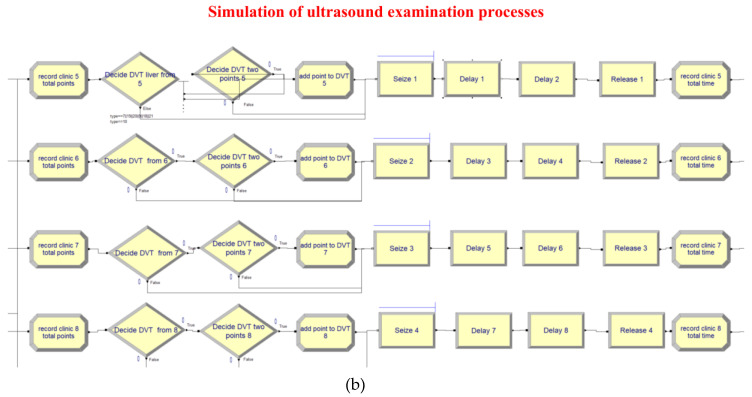
(**a**) Simulation of the logic for assigning patients to the ultrasound examination rooms. (**b**) Simulation of the ultrasound examination processes.

In Figure 4a,b, the simulation model for assigning the patients to the ultrasound examination rooms was constructed based on the flow chart of Figure 2. Outpatients, inpatients, and emergency patients entered the system according to their respective times that exhibited empirical distribution. The examination time and body part code generated using the examination time distributions were then assigned to patients undergoing ultrasound examination to determine subsequent examination time distribution and examination room assignment. The simulated examination process was confirmed to be consistent with the actual process, and the examination room assignment logic of the simulation model was consistent with that set by this study; the simulation model was deemed to pass the verification test.

(2) Validation

After verifying the logic correctness of the simulation model, this study compared the actual average total number of arrivals of the three types of patients every 5 days and the proportion of patients for each body part examined with those in the simulation model to confirm whether any significant difference existed between the simulation model and the actual system. According to the data provided by the hospital, the actual average total number of outpatients in the examination rooms was 355.3, which was within the 95% confidence interval, or [349.8, 356.8], of the simulation model. The actual average total number of inpatients in the examination rooms was 93.0, which was within the 95% confidence interval, or [91.9, 95.5], of the simulation model. The actual average total number of emergency patients at the examination rooms was 37.9, which was within the 95% confidence interval, or [35.8, 38.2], of the simulation model. Therefore, no significant difference existed between the simulated number of arrivals for each type of patients every 5 days and that in the actual system.

To validate the simulation regarding the number of outpatients undergoing ultrasound examination of various body parts, this study confirmed whether the actual average total number of patients per week was within the 95% confidence interval for the simulated average total number of patients per week. Using the data regarding outpatients undergoing the abdomen ultrasound examination as an example, the actual average total number of patients per week was 135.3, which was within the 95% confidence interval, or [133.2, 137.5], of the simulation model. The number of patients undergoing ultrasound examination of the remaining body parts was also within the 95% confidence interval. Taking the same approach, this study found that the actual average total number of inpatients and emergency patients undergoing ultrasound examination of various body parts was also within the 95% confidence interval. Therefore, no significant difference existed between the number of patients undergoing ultrasound examination of various body parts in the simulation model and the actual number of patients.

According to this validation, the number of patients entering the actual system and the actual number of patients undergoing ultrasound examination of various body parts were within the 95% confidence interval for the simulated data in the simulation model. Thus, no significant difference existed between the simulation model constructed in this study and the actual situation in the radiology department of the hospital. This indicates that the simulation model passed the validation test, and can represent the performance of the actual system to perform scenario analysis.

### 4.7. Scenario Analyses

This study investigated three scenarios with different directions for improvement to improve the current situation (AS-IS model) of the radiology department in the case hospital. The appointment scheduling time interval for patients undergoing the ultrasound examination under the AS-IS model represented the actual arrival status of patients undergoing ultrasound examination in the hospital. The cumulated examination points were used as the basis for assigning patients to the examination rooms. Scenario 1 explored changes in the time interval for the arrival of outpatients at the radiology department on the examination day. Scenario 2 explored changes on the basis of determining the assignment of patients undergoing the ultrasound examination. This scenario was divided into two stages: The first stage did not consider the operational proficiency of radiological technologists, whereas the second stage did. Scenario 3 explored changes in the time intervals for the arrival of patients undergoing the ultrasound examination at the radiology department on the examination day and the basis for determining the assignment of patients to the examination rooms.

In Scenario 1, the waiting time for the patients undergoing the ultrasound examination and the equipment utilization rate on the examination day were adopted as performance indicators to determine the best appointment time interval. The hospital limits the workload of radiological technologists in all examination rooms to between 70% and 75%; moreover, the total number of patients examined must be greater than 485 (the original total number of patients examined). Therefore, this study constructed an optimized simulation model, and employed the simulation software, Arena with OptQuest, to determine the best appointment time interval that met the expectations of the case hospital.

To minimize the average patient waiting time, the objective function was set as the number of each type of patient multiplied by their waiting time, then divided by the total number of patients to obtain the solution for minimizing their average waiting time. Regarding constraints, after discussion with the hospital, the equipment utilization rate in each examination room was limited to more than 70%, and the total number of arriving patients was greater than 485 in the actual situation.

**Figure 5:**
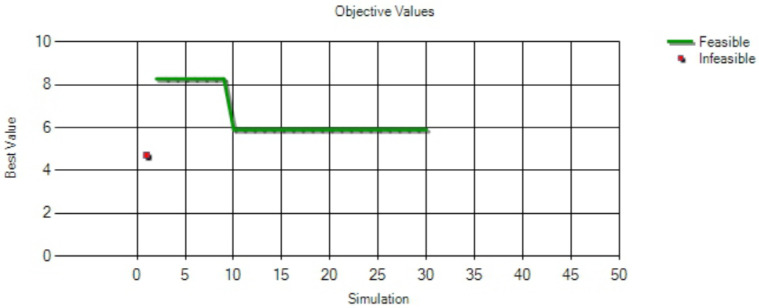
The results of optimization performed by the simulation model at different time intervals.

Figure 5 shows the results of optimization performed by the simulation model under different time intervals for the arrival of outpatients undergoing the ultrasound examination, which ranged from 1 to 30 min. Arena with OptQuest found the best solution among the feasible solutions from the algorithm after the 10th iteration (or simulation).

**Figure 6:**
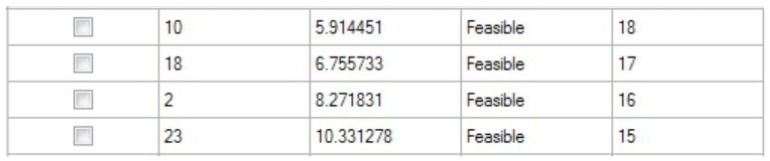
The best four feasible solutions after optimization.

Figure 6 shows the four feasible solutions obtained after optimization, ranked in an ascending order according to the minimum value of the objective function. When the time interval for the arrival of outpatients undergoing the ultrasound examination was less than or equal to 18 min, all the four solutions satisfied the constraint conditions. The best time interval for the arrival of outpatients undergoing ultrasound examination was 18 min, which minimized the average waiting time for patients undergoing ultrasound examination, where the minimum average waiting time was 5.91 min.

Table 3 compares the actual situation and the implementation results when the best time interval was 18 min. This table shows the average total number of patients examined was 504.04, and the equipment utilization rates in all the examination rooms were greater than 70%, which satisfied the constraints in this study. In addition, the average total number of patients obtained from the best solution was approximately 20 people more than the average total number of patients under the AS-IS model. The best solution for the average waiting time for patients undergoing ultrasound examination was 1.32 min less than the average waiting time for patients under the AS-IS model.

In Scenario 2, this study simulated changes on the basis of determining the assignment of patients to examination rooms, and its performance indicators were the waiting time for patients undergoing ultrasound examination on the examination day, the workload of radiological technologists, and the SD of the workload of radiological technologists. This study investigated three models to determine the assignment of patients undergoing ultrasound examination to examination rooms: the AS-IS model, which used cumulative examination points; the TO-BE1 model, which used cumulative examination time; and the TO-BE2 model, which used random assignment.

Table 4 shows that the waiting time for patients undergoing ultrasound examination on the examination day decreased by 2.98 min under the TO-BE1 model, compared with that under the AS-IS model. In addition, the SD of the workload of radiological technologists under the TO-BE1 model was 0.06%, which was less than the 1.40% under the AS-IS model. Furthermore, this study used the SD of the workload of radiological technologists as the basis for determining whether the workload of radiological technologists was balanced, where the lower the value, the more balanced the workload of radiological technologists. Therefore, the workload of radiological technologists was more balanced under the TO-BE1 model.

Compared with the AS-IS model, the waiting time for patients undergoing ultrasound examination on the examination day increased by 14.70 min under the TO-BE2 model. Moreover, the SD of the workload of radiological technologists under the TO-BE2 model was 13.69%, which was greater than that under the AS-IS model at 1.40. Thus, the best model obtained in the second stage in Scenario 2 was the TO-BE1 model.

Despite the TO-BE2 model significantly reducing the SD of the workload of radiological technologists, the director of the radiology department believed that each radiological technologist’s equipment operation proficiency was different. If the cumulative examination time were to be used as the basis for assigning patients to examination rooms, it may lead to a situation in which highly proficient radiological technologists work at a faster pace due to their experience in operating the equipment, and thus take a shorter time for each examination. Consequently, highly proficient radiological technologists may examine more patients than those with less proficiency.

The director of the radiology department in the case hospital hoped to develop a fair method for the number of patients examined and the examination time. Therefore, this study suggested using the weighted cumulative examination points, and assigning weighted points to the average examination time for each body part calculated in this study according to the baseline of 20 min. For instance, the actual average examination time for outpatients undergoing shoulder ultrasound examination was 16.35 min. Therefore, 16.35 min divided by 20 min, or 0.8 points, were assigned to this value of average examination time. However, the time between a patient’s entry into the examination room and the upload of the first ultrasound image was approximately 5 min, and thus 5 min divided by 20 min, or 0.3 points, were assigned to this value of time. Therefore, the total number of weighted points was 1.1 points.

When the three models were investigated during the second stage of Scenario 2, the AS-IS model used the cumulative examination points as the basis for determining the assignment of patients undergoing ultrasound examination to examination rooms; the TO-BE1 model used the cumulative examination time, whereas the TO-BE3 model used the weighted cumulative examination points.

As shown in Table 5, the waiting time for patients undergoing the ultrasound examination on the examination day increased by 1.01 min under the TO-BE3 model, compared with that under the TO-BE1 model. Furthermore, the SD of the workload of radiological technologists under the TO-BE3 model was 0.54%, which was greater than the SD of the workload under the TO-BE1 model at 0.06%, because the workload under the TO-BE3 model was less balanced than that under the TO-BE1 model. Compared with the AS-IS model, the waiting time for patients undergoing ultrasound examination on the examination day decreased by 1.97 min under the TO-BE3 model. Additionally, the SD of radiological technologists’ workload under the TO-BE3 model was 0.54%, which was less than that under the AS-IS model at 1.40. Therefore, the workload of radiological technologists under the TO-BE3 model was more balanced than that under the AS-IS model. Although the TO-BE3 model did not produce superior results to the TO-BE1 model, the results obtained under the TO-BE3 model met the expectations of the hospital’s radiology department director because using the weighted cumulative examination points reduced the unfair situation associated with the equipment operation proficiency of radiological technologists. The TO-BE3 model also produced superior results to the AS-IS model. Thus, the most practical and feasible model was the TO-BE3 model in the second stage of Scenario 2.

In Scenario 3, this study simulated changes both in the time interval for the arrival of outpatients undergoing ultrasound examination at the radiology department on the examination day and in the basis for determining the assignment of patients to examination rooms. The waiting time for patients, equipment utilization rate, and SD of radiological technologists’ workload were adopted as performance indicators. Two models were investigated in this scenario. The AS-IS model indicated the actual arrival status of patients undergoing ultrasound examination (where 4.7 patients arrive at the radiology department every 20 min on average), and used the cumulative examination points as the basis for determining the assignment of patients to examination rooms. Under the TO-BE4 model, three outpatients arrived at the radiology department every 18 min, and the number of inpatients and emergency patients arriving at the radiology department was determined based on the original distribution of time intervals. In addition, this solution used the weighted cumulative examination points as the basis for determining the assignment of patients to examination rooms.

As shown in Table 6, the waiting time for patients undergoing ultrasound examination on examination day decreased by 3.12 min under the TO-BE4 model, compared with that under the AS-IS model. The SD of the workload of radiological technologists under the TO-BE4 model was 0.39%, which was less than that under the AS-IS model at 1.40%; thus, the workload of radiological technologists under the TO-BE4 model was more balanced than that under the AS-IS model. The best model in Scenario 3 was TO-BE4.

### 4.8. Discussions

This study used a simulation model to simulate three scenarios, and discovered that the length of the time interval for the arrival of patients at the radiology department affected the number of patients undergoing ultrasound examination and in turn affected the waiting time on the examination day and the workload of radiological technologists. Analysis of the three scenarios suggests that in Scenario 1, the best time interval for the arrival of outpatients undergoing ultrasound examination is 18 min. In the first stage of Scenario 2, the best model is to use cumulative examination time as the basis for determining the assignment of patients to examination rooms. In the second stage of Scenario 2, the best model is to use the weighted cumulative examination points as the basis for determining the assignment of patients to examination rooms. In Scenario 3, this study recommends that three outpatients arrive at the radiology department for the ultrasound examination every 18 min, and the number of inpatients and emergency patients undergoing ultrasound examination are consistent with the original time interval distribution. Moreover, the best model in Scenario 3 is to use the weighted cumulative examination points as the basis for determining the assignment of patients undergoing ultrasound examination to examination rooms. These models offered high performance in terms of waiting time for patients undergoing ultrasound examination on the examination day, equipment utilization rate, and SD of the workload of radiological technologists.

When it comes to scheduling and room assignment for patients, the findings of this research may be adapted by hospital management into policies and then incorporated into the information management system used by the radiology department.

In future work, radiologist or staff experience may also be considered since their proper training and proficiency may affect the diagnosis and length of time needed for examination.

The work has a number of limitations. The hospital investigated in this study had only six examination rooms (i.e., Examination Rooms 5 to 10). Further, in accordance with the radiology department’s policy, inpatients were given priority to undergo ultrasound examination in Examination Room 5, while emergency patients were given priority to undergo ultrasound examination in Examination Room 6.

## 5. Conclusions

Previous studies have discussed patient appointment scheduling and the methods for assigning patients to examination rooms at hospitals; however, they have not discussed how these may affect patients’ waiting time and radiological technologists’ workload. The study makes the following contributions to the literature: This study investigated the aforementioned issues through system simulation and used Arena to construct a simulation model. It also employed Arena with OptQuest to find the best appointment scheduling time interval for multiple examination rooms, multiple types of patients, and ultrasound examination of multiple body parts. This study used these simulation models to investigate the best examination room assignment strategy under the conditions of multiple examination rooms, multiple types of patients, and examination of multiple body parts in different scenarios. Moreover, this study used these simulation models to jointly investigate the time intervals in the appointment scheduling problem for patients undergoing ultrasound examination and the assignment of the patients to examination rooms. This study provided best models under different scenarios that met the expectations of the radiology department in the case hospital. These models maintained high equipment utilization rates, achieved a balanced workload for radiological technologists, and reduced patient waiting times.

This study investigated the appointment scheduling and examination room assignment problems for patients undergoing ultrasound examination under the conditions of multiple types of patients, multiple examination rooms, and examination of multiple body parts. However, in practice, there could be no-show patients even after appointments are made [9,22]. Therefore, future studies are recommended to consider this limitation. Furthermore, the examination rooms in the radiology department of the hospital investigated in this study used equipment with identical functions. However, equipment with heterogeneous functions may be used in other hospitals [1]; therefore, future studies can include this constraint and use scenarios in other hospitals to find better solutions regarding the appointment scheduling time intervals for patients undergoing ultrasound examination and the assignment of the patients to examination rooms. Further development can include priority-based routing strategies for the examination room assignment problems. Moreover, different simulation and decision-making methodologies, including system dynamics [11] and AHP approaches [10], can be employed to evaluate the most efficient model.

## Figures and Tables

**Table 1:** Literature review on outpatient appointment scheduling problems and operating room scheduling problems.

References	Problem	Mathematical Model or Simulation Model	Methodology
Chen et al. [1]	Outpatient appointment scheduling	Mathematical model and discrete-event simulation model	System simulation with simulation optimization
Sun et al. [5]	Outpatient appointment scheduling	Mathematical model	Stochastic programming and heuristic algorithms
Qiu et al. [6]	Outpatient appointment scheduling	Mathematical model	Meta-heuristic algorithms
Cappanera et al. [8]	Outpatient appointment scheduling	Mathematical model	Heuristic algorithms
Pan et al. [14]	Outpatient appointment scheduling	Mathematical model	Stochastic programming and heuristic algorithms
Millhiser and Veral [15]	Outpatient appointment scheduling	Mathematical model and discrete-event simulation model	System simulation
Ahmed and Alkhamis [17]	Operating room scheduling	Mathematical model	System simulation with simulation optimization
Rau et al. [18]	Outpatient physical therapy service	Discrete-event simulation model	System simulation
Hur et al. [19]	Outpatient appointment scheduling	Mathematical model	Stochastic programming and heuristic algorithms
Huang et al. [20]	Operating room scheduling	Monte Carlo simulation	System simulation
Lee et al. [23]	Outpatient appointment scheduling	Mathematical model	Heuristic algorithms
Klassen and Yoogalingam [26]	Outpatient appointment scheduling	Mathematical model	Simulation optimization
Moreno and Blanco [30]	Outpatient appointment scheduling	Mathematical model	Mathematical software
Baesler et al. [31]	Operating room scheduling	Discrete-event simulation model	System simulation
Xiao et al. [35]	Operating room scheduling	Mathematical model	Stochastic programming and sample average approximation method
Baril et al. [38]	Outpatient orthopaedic clinic	Discrete-event simulation model	System simulation
Wu et al. [40]	Outpatient appointment scheduling	Discrete-event simulation model	System simulation
Liu [43]	Outpatient appointment scheduling	Mathematical model	Queueing theory
Ma et al. [44]	Outpatient appointment scheduling	Mathematical model	Heuristic algorithms
Ferrand et al. [48]	Operating room scheduling	Discrete-event simulation model	System simulation

**Table 2:** Proportion of Patient Category at the Radiology Department.

Patient Category	Number of Patients	Percentage	Cumulative Percentage
Outpatient	10,133	72.46%	72.46%
Inpatient	2794	19.98%	92.43%
Emergency Patients	1058	7.57%	100.00%
Total	13,985	100.00%	

**Table 3:** The comparison results of actual situation and implementation results at the best time interval of 18 min.

Performance Indicator	AS-IS Model	OptQuest Best Model
Patients’ arrival interval at the radiology department (Minute)	Empirical Value	18
Average total number of examined patients (Person)	484.00	504.04
Patient’s average waiting time (Minute)	7.23	5.91
Radiologist’s workload at the 5th examination room (equipment utilization rate) (%)	71.39	74.62
Radiologist’s workload at the 6th examination room (equipment utilization rate) (%)	69.97	73.17
Radiologist’s workload at the 7th examination room (equipment utilization rate) (%)	69.44	72.37
Radiologist’s workload at the 8th examination room (equipment utilization rate) (%)	68.02	70.28
Radiologist’s workload at the 9th examination room (equipment utilization rate) (%)	68.93	72.15
Radiologist’s workload at the 10th examination room (equipment utilization rate) (%)	67.49	70.88

**Table 4:** Execution results between AS-IS and TO-BE models at the Stage 1 of Scenario 2.

Performance Indicator	AS-IS Model		TO-BE1 Model		TO-BE2 Model	
	Average	The Half-Width of 95% Confidence Interval	Average	The Half-Width of 95% Confidence Interval	Average	The Half-Width of 95% Confidence Interval
Average total number of examined patients (Person)	484.00	2.98	487.24	3.12	487.81	2.98
Patient’s average waiting time (Minute)	7.23	0.35	4.25	0.24	21.93	0.94
Radiologist’s workload at the 5th examination room (equipment utilization rate) (%)	71.39	0.01	69.68	0.01	87.74	0.01
Radiologist’s workload at the 6th examination room (equipment utilization rate) (%)	69.97	0.01	69.55	0.01	82.74	0.01
Radiologist’s workload at the 7th examination room (equipment utilization rate) (%)	69.44	0.01	69.52	0.01	54.73	0.02
Radiologist’s workload at the 8th examination room (equipment utilization rate) (%)	68.02	0.01	69.56	0.01	68.87	0.02
Radiologist’s workload at the 9th examination room (equipment utilization rate) (%)	68.93	0.01	69.59	0.01	55.06	0.01
Radiologist’s workload at the 10th examination room (equipment utilization rate) (%)	67.49	0.01	69.60	0.01	67.92	0.01
Radiologist’s average workload (%)	69.21	-	69.58	-	69.51	-
SD of radiologist’s workload (%)	1.40	-	0.06	-	13.69	-

**Table 5:** Execution results between AS-IS and TO-BE models in the Stage 2 of Scenario 2.

Performance Indicator	AS-IS Model		TO-BE1 Model		TO-BE3 Model	
	Average	The Half-Width of 95% Confidence Interval	Average	The Half-Width of 95% Confidence Interval	Average	The Half-Width of 95% Confidence Interval
Average total number of examined patients (person)	484.00	2.98	487.24	3.12	480.94	2.83
Average patient’s waiting time (minute)	7.23	0.35	4.25	0.24	5.26	0.27
Radiologist’s workload at the 5th examination room (equipment utilization rate) (%)	71.39	0.01	69.68	0.01	68.35	0.01
Radiologist’s workload at the 6th examination room (equipment utilization rate) (%)	69.97	0.01	69.55	0.01	68.00	0.01
Radiologist’s workload at the 7th examination room (equipment utilization rate) (%)	69.44	0.01	69.52	0.01	69.42	0.01
Radiologist’s workload at the 8th examination room (equipment utilization rate) (%)	68.02	0.01	69.56	0.01	68.69	0.01
Radiologist’s workload at the 9th examination room (equipment utilization rate) (%)	68.93	0.01	69.59	0.01	69.20	0.01
Radiologist’s workload at the 10th examination room (equipment utilization rate) (%)	67.49	0.01	69.60	0.01	69.09	0.01
Radiologist’s average workload (%)	69.21	-	69.58	-	68.79	-
SD of radiologist’s workload (%)	1.40	-	0.06	-	0.54	-

**Table 6:** Execution results between AS-IS and TO-BE models in Scenario 3.

Performance Indicator	AS-IS Model		TO-BE4 Model	
	Average	The Half-Width of 95% Confidence Interval	Average	The Half-Width of 95% Confidence Interval
Average total number of examined patients (person)	484.00	2.98	505.42	0.42
Patient’s average waiting time (minute)	7.23	0.35	4.11	0.22
Radiologist’s workload at the 5th examination room (equipment utilization rate) (%)	71.39	0.01	72.11	0.01
Radiologist’s workload at the 6th examination room (equipment utilization rate) (%)	69.97	0.01	71.75	0.01
Radiologist’s workload at the 7th examination room (equipment utilization rate) (%)	69.44	0.01	72.78	0.01
Radiologist’s workload at the 8th examination room (equipment utilization rate) (%)	68.02	0.01	72.54	0.01
Radiologist’s workload at the 9th examination room (equipment utilization rate) (%)	68.93	0.01	72.67	0.01
Radiologist’s workload at the 10th examination room (equipment utilization rate) (%)	67.49	0.01	72.24	0.01
Radiologist’s average workload (%)	69.21	-	72.35	-
SD of radiologist’s workload (%)	1.40	-	0.39	-
